# Green Synthesis of Hierarchically Structured Silver-Polymer Nanocomposites with Antibacterial Activity

**DOI:** 10.3390/nano6080137

**Published:** 2016-07-25

**Authors:** María Jesús Hortigüela, Luis Yuste, Fernando Rojo, Inmaculada Aranaz

**Affiliations:** 1 Instituto de Ciencia de los Materiales, Consejo Superior de Investigaciones Científicas, Campus of Cantoblanco, Madrid 28049, Spain; mjhortiguela@gmail.com; 2 Centro Nacional de Biotecnología, Consejo Superior de Investigaciones Científicas, Campus of Cantoblanco, Madrid 28049, Spain; lyuste@cnb.csic.es (L.Y.); frojo@cnb.csic.es (F.R.)

**Keywords:** chondroitin sulfate, nanoparticles, silver, scaffolds

## Abstract

The in situ formation of silver nanoparticles (AgNPs) aided by chondroitin sulfate and the preparation of a hierarchically structured silver-polymer nanocomposite with antimicrobial activity is shown. Green synthesis of AgNPs is carried out by thermal treatment (80 and 90 °C) or UV irradiation of a chondroitin sulfate solution containing AgNO_3_ without using any further reducing agents or stabilizers. Best control of the AgNPs size and polydispersity was achieved by UV irradiation. The ice-segregation-induced self-assembly (ISISA) process, in which the polymer solution containing the AgNPs is frozen unidirectionally, and successively freeze-drying were employed to produce the chondroitin sulfate 3D scaffolds. The scaffolds were further crosslinked with hexamethylene diisocyanate vapors to avoid water solubility of the 3D structures in aqueous environments. The antimicrobial activity of the scaffolds was tested against *Escherichia coli*. The minimum inhibitory concentration (MIC) found for AgNPs-CS (chondroitin sulfate) scaffolds was ca. 6 ppm.

## 1. Introduction

The synergy among entities of a different nature that is found in nanocomposites will perform a key function in the progress of advanced functional materials in diverse fields, mainly in biomedical applications [1]. Keeping in mind the strict biocompatibility requirements of biomedical devices, the use of green methods for nanoparticle preparation is crucial [2]. Otherwise, detrimental effects on biological entities can easily occur due to the use, during and/or post synthesis, of non-biocompatible chemical reagents such as reducing agents, residual solvents or surfactants among others which, in the end, may be difficult to remove from the reaction media by practical manufacturing techniques. Furthermore, the assembly of nanoparticles into macroscopic materials is attracting much attention [3]. Thus, progress in this field depends largely on the core competence of materials scientists to create new synthetic approaches (mainly derived from bottom-up techniques) for the development of advanced materials with nano- and microstructures and unmatched achievements [4,5,6]. Thus, we should not only be able to produce nanostructures, controlling both their shape and size, but also to build them in any appearance and to produce hierarchically organized structures so that accessibility to the inner interfaces is guaranteed [7].

Any green synthetic approach needs to consider the use of harmless chemicals and solvents, environmentally safe reducing agents and renewable materials. Moreover, the in situ preparation of the nanocomposites into the demanded structure (e.g., nanoparticles, particles, fibers, capsules, scaffolds, etc.) can also be of help to further enhance the overall biocompatibility of the preparation process by reduction of manipulation steps that may finally lead to contamination [8].

Silver nanoparticles (AgNPs) have optical, antimicrobial and electrical properties that depend both on their size and shape. Ag^+^ reduction to Ag nanoparticles can be carried out by chemical methods using reducing agents such as glucose, sodium borohydrate, ascorbic acid, etc., by electrochemical methods via photochemical processes, or by radiation [9]. In a first step, silver atoms (Ag^0^) are reduced to silver ions (Ag^+^), which is followed by agglomeration into oligomeric clusters, and finally ends in the formation of colloidal Ag particles. Due to the tendency of the AgNPs to aggregate, it is necessary to use capping agents to stabilize the AgNPs. 

**Figure 1:**
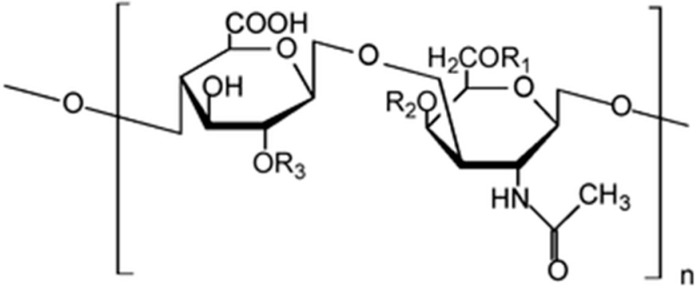
Chemical structure of chondroitin-4-sulfate, where R_1_ = H; R_2_ = SO_3_H; R_3_ = H.

Several examples of AgNPs synthesis in the presence of naturally occurring stabilizers such as cellulose, chitosan, heparin and starch, among others, have been reported [10,11,12]. The use of chondroitin sulfate (CS) to produce AgNPs has also been reported, with the polymer acting both as a reducing and stabilizing agent by capping the AgNPs [13]. CS is a sulfated glycosaminoglycan (Figure 1) that has attracted great interest as a biocompatible and biodegradable polymer employed in pharmaceutical and biomedical devices. CS has been applied in cartilage regeneration, bone regeneration, skin regeneration or neuronal regeneration [14]. Recently, 3D nanocomposites composed of chondroitin sulphate and multi-walled carbon nanotubes (MWCNTs) for nervous tissue repair have been described [15].

The management of infections associated with surgical implants can be both complicated and costly. Therefore, the prevention of such infections by developing medical devices containing antimicrobial agents is a topic of great interest.

AgNPs thermally produced in the presence of chondroitin sulfate and their use in an ointment with antibacterial properties and in vivo wound healing activity has been recently reported [16], but to our knowledge, the green synthesis of AgNPs-CS three-dimensional nanocomposites with antibacterial activity has not been reported.

## 2. Results and Discussion

### 2.1. AgNPs Production and Characterization

In this paper, we report the green synthesis of 3D structured hierarchically nanocomposites, in particular of silver nanoparticles entrapped within chondroitin sulfate scaffolds (AgNPs-CS scaffolds).

**Figure 2:**
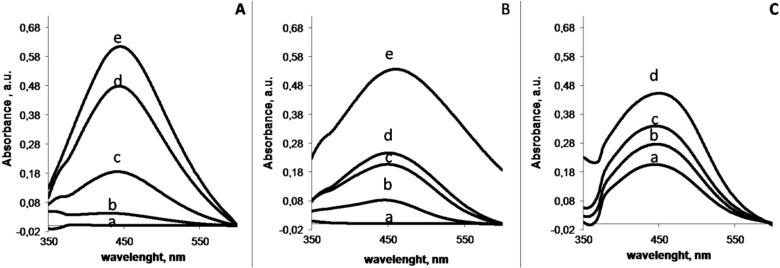
UV-Vis spectra of silver-containing chondroitin sulphate solutions (CS 10 wt. % and 0.16 mM AgNO_3_) after thermal treatment at 80 °C (**A**) and 90 °C (**B**) and UV-treatment (**C**). Thermal treatments were performed over 0, 90, 120, 210 and 270 min (lines a, b, c, d and e, respectively). UV treatments were performed over 120, 180 and 240 min (lines a, b and c). Sample irradiated during 240 min rested 24 h before measuring the exciton peak (line d).

AgNPs were obtained by reduction of silver salts in an aqueous solution of chondroitin sulfate by thermal treatments (80 and 90 °C) or by UV light irradiation [17]. During the different treatments, a yellow color appeared in the solutions over time, indicating the formation of AgNPs. The intensity of the silver exciton peak at 400 nm [18] was used as an indicator of nanoparticle synthesis and growth, as seen in Figure 2.

The band broadening and red shift shown in the spectra of the AgNPs produced by the thermal treatment (90 °C) point to large AgNPs and wide size distribution, and therefore this method was rejected for producing the AgNPs. As seen in Figure 2A,C, AgNPs produced by thermal treatment (80 °C) and UV irradiation showed narrower spectra centered at 445 nm. Interestingly, the intensity of the AgNPs exciton increased after resting the UV-irradiated solution 24 h in the dark before the measurement.

As controls, AgNO_3_ solutions without polymer were also thermally treated or irradiated and the absorbance of the solution was monitored over time. In both cases, no AgNPs formation was detected under the experimental conditions.

**Figure 3:**
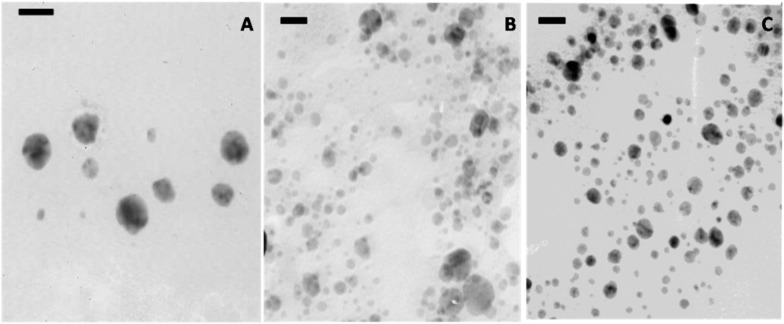
Transmission electron microscopy (TEM) micrographs of AgNPs-CS produced by thermal treatment at 80 °C (**A**); UV irradiation of fresh sample (**B**); and sample rested for 24 h (**C**). Bars are 50 nm in panel (**A**) and 20 nm in panels (**B**) and (**C**). CS: chondroitin sulfate.

AgNPs were spherical in shape and the samples produced by the thermal treatment were larger than those produced by UV irradiation (Figure 3). The 24 h resting period seems to be necessary for proper AgNPs formation after UV treatment as seen in Figure 3B,C.

**Figure 4:**
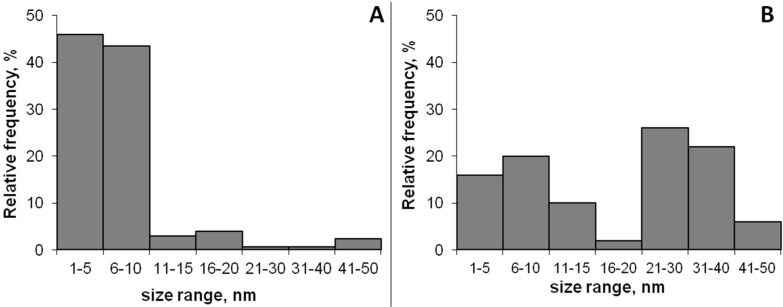
Particle size distribution estimated from TEM micrographs of AgNPs produced by UV treatment (**A**) and by thermal treatment (**B**). *N* =100.

Transmission electron microscopy (TEM) micrographs were used to determine the AgNPs’ size distribution; at least 100 AgNPs were analyzed using Image J software. As seen in Figure 4, more than 90% of the AgNPs produced by UV irradiation had a particle size lower than 10 nm while the AgNPs produced by thermal treatment (80 °C) had a bimodal distribution with particle sizes centered in the range of 1–10 nm and 20–40 nm, respectively.

**Figure 5:**
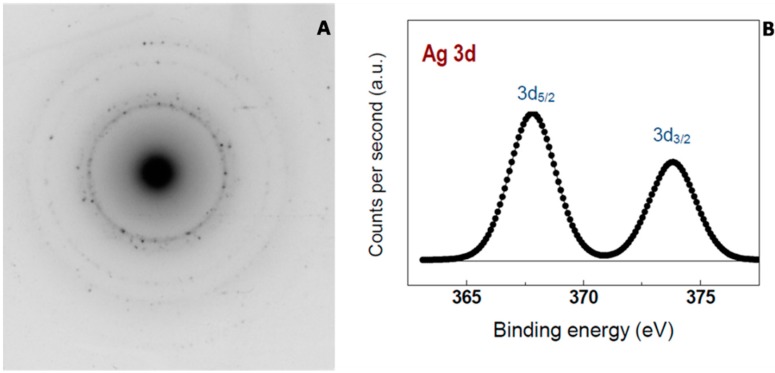
Metallic nature of AgNPs. Diffraction pattern (**A**) and X-ray photoelectron spectroscopy (XPS) spectrum (**B**) of the AgNPs produced by UV irradiation.

The metallic nature of the AgNPs was corroborated by selected-area electron diffraction (SAED) and X-ray photoelectron spectroscopy (XPS) (Figure 5). The corresponding SAED pattern showed the main diffraction rings that can be assigned as (111), (200), (220) and (311) reflections, which corresponds to the face-centered cubic structure of metallic silver. Moreover, the XPS analysis revealed the presence of Ag 3d_5/2_ and Ag 3d_3/2_ doublet peaks typical of metallic silver at binding energies of 367.8 and 373.8 eV, respectively, with a spin-orbit separation of 6.0 eV. The yield of the reduction process was around 100% since no Ag^+^ was detected.

### 2.2. AgNPs-CS Scaffold Preparation and Characterization

It has been well recognized since ancient times that metallic silver displays antimicrobial activities, but its use was relegated by modern antibiotics in the last decades [19]. The emergence of bacterial resistances to frequently used antibiotics has been a key factor in the considerable attention that AgNPs have gained in the last years. In general, the smaller the particle size is, the greater the antimicrobial effect is. This can be explained by the fact that small size implies large available surface areas, higher silver ion release and improved interactions with other particles [20,21]. Taking this into account, we selected the AgNPs produced by UV irradiation to produce the AgNPs-CS scaffolds. The resulting irradiated polymer solution containing AgNPs was submitted to the ice-segregation-induced self-assembly (ISISA) process for the formation of a macroporous monolith [22]. Because CS is highly soluble in water, the scaffold was subsequently crosslinked with hexamethylene diisocyanate vapors at 60 °C over one week to avoid its dissolution in aqueous media.

**Figure 6:**
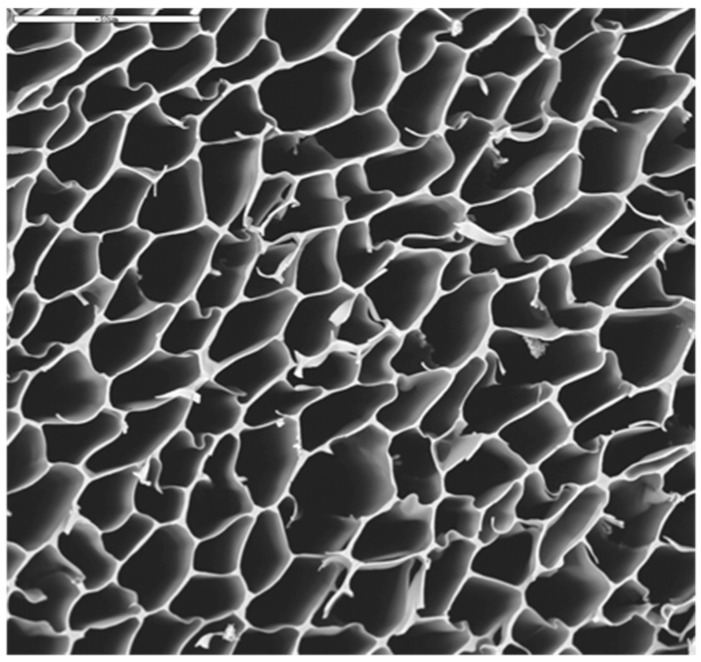
Scanning electron microscopy (SEM) micrograph of AgNPs-CS crosslinked scaffold. Bar 50 µm.

As confirmed by Scanning electron microscopy (SEM), the resulting crosslinked scaffold exhibited a uniform and porous 3D structure along the whole scaffold, with a pore size range between 10 and 30 μm (Figure 6). The Ag content within the scaffolds was 287 ± 13 µg of Ag per gram of polymer.

### 2.3. Silver Release from AgNPs-CS Crosslinked Scaffolds

**Figure 7:**
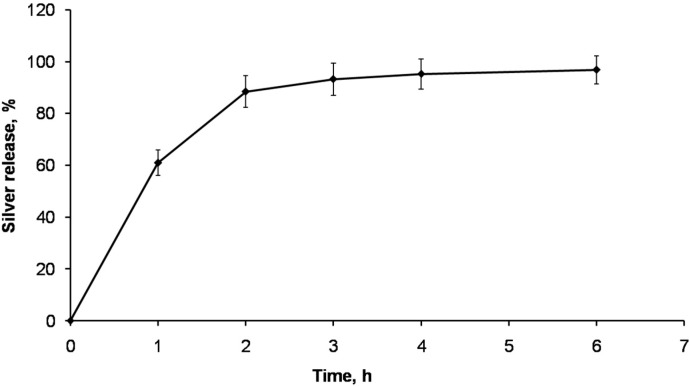
Silver release from AgNPs-CS crosslinked scaffolds in phosphate saline buffer (PSB) at 37 °C. *N* =2.

Since the antimicrobial properties of the scaffold will depend on its ability to release silver to the media, the silver release from the AgNPs-CS crosslinked scaffolds was studied in physiological conditions (phosphate saline buffer (PSB) at 37 °C). As seen in Figure 7, the release of the silver from the scaffolds was very fast, with around 60% of the silver released from the scaffolds in the first hour. Moreover, after 6 h more than 95% of the silver was released from the scaffold. This release pattern is in good agreement with the silver release from a hydrophilic matrix, in which the hydrophilic polymeric matrix highly swells in aqueous media, allowing a fast release of the antimicrobials from the matrix [23]. Therefore, the minimum inhibitory concentration is largely exceeded in a short time, helping to prevent post-surgical infections.

### 2.4. AgNPs-CS Antimicrobial Activity

**Figure 8:**
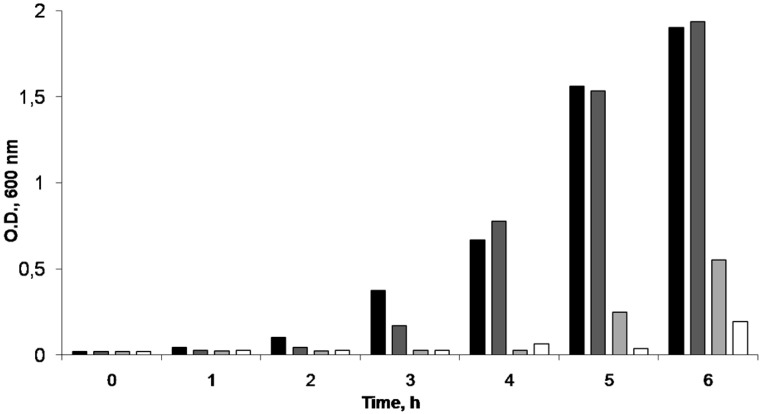
*E. coli* growth inhibition for different Ag concentrations. Ag 0 ppm (■); Ag 1.32 ppm (■); Ag 3.76 ppm (■); Ag 6.26 ppm (□).

The antimicrobial activity of the AgNPs entrapped in the CS scaffolds was studied. To ascertain the minimum inhibitory concentration of the AgNPs, we dissolved a known amount of a non-crosslinked scaffold in aqueous media and different aliquots were produced by dilution in PBS. The antimicrobial activity of each dilution against *E. coli* was tested. As seen in Figure 8, the minimum inhibitory concentration (MIC) found for the AgNPs was ca. 6.2 ppm, in good agreement with data previously reported [24,25]. When a bare scaffold (sample without silver) was tested (sample denoted as 0 ppm in Figure 8), *E. coli* grew in the culture media. That means that CS did not exert any antimicrobial activity and the observed antimicrobial activity of the AgNPS-CS scaffolds is only ascribed to the AgNPs incorporated in the scaffold.

**Figure 9:**
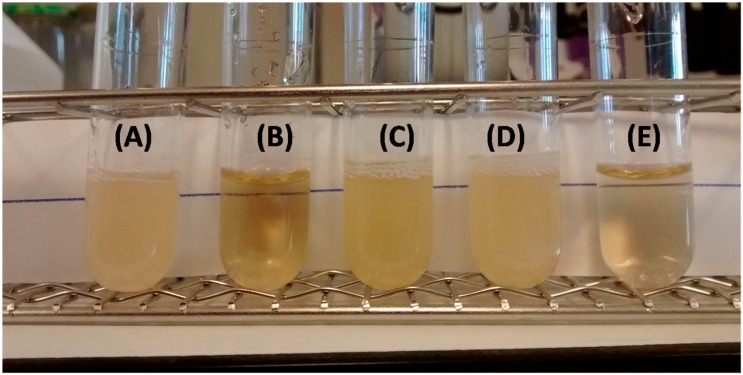
Antimicrobial activity of CS crosslinked scaffold (**A**); AgNPs-CS crosslinked scaffold (**B**); AgNO_3_ crosslinked scaffold (**C**); AgNO_3_ aqueous solution (Ag^+^: 6.25 ppm) (**D**), and AgNO_3_ aqueous solution (Ag^+^: 12.5 ppm).

The antibacterial activity of AgNp-CS crosslinked scaffolds, AgNO_3_-CS crosslinked scaffolds and CS crosslinked scaffolds was tested against *E. coli* at 24 h (Figure 9A–C). In all cases, the same amount of scaffolds (ca. 60 mg) was used; this amount was selected taking into account the silver content of the scaffolds and guarantees a silver concentration in the culture media larger than the MIC concentration (around 8 ppm).

The antimicrobial activity was merely detected in the media containing AgNPs-CS crosslinked scaffolds, while no antimicrobial activity was found when the same amount of AgNO_3_-CS crosslinked scaffold or CS crosslinked scaffold were tested. The lack of antimicrobial activity of the CS crosslinked scaffold demonstrated that the antimicrobial activity observed in the AgNPs-CS crosslinked scaffolds was not due to hexymethylene diisocyanate toxicity. Interestingly, no antimicrobial activity was detected when AgNO_3_-CS crosslinked scaffolds were tested. To further investigate the antimicrobial activity of AgNO_3_, two aqueous solutions of AgNO_3_ (6.25 and 12.5 ppm) were tested. No antimicrobial activity was observed at 6.25 ppm while no bacterial growth was observed at 12.5 ppm (Figure 9D,E). This indicated that AgNPs produced by UV irradiation of a chondroitin sulfate solution have better antimicrobial activity than AgNO_3_. The precise antimicrobial mechanism of silver is still not known, but different mechanisms of action have been suggested, depending on the ionized state of silver, which may explain the different behavior observed between AgNPs and silver nitrate [26].

## 3. Materials and Methods

### 3.1. Materials

AgNO_3_ with a purity >99.5.0% was purchased from Fluka. Chondroitin 4-sulfate sodium salt (CS) from bovine trachea was purchased from Sigma. Ultrapure water obtained with a Milli-Q equipment (Millipore, Billerica, MA, USA) was used as solvent.

### 3.2. Methods

#### 3.2.1. Preparation of Ag Nanoparticles in Chondroitin Sulfate Solution by Thermal Treatment

First 400 µL of AgNO_3_ (1 mM) dissolved in water were added to a chondroitin sulfate solution (10% *w*/*v*) and then the mixture was thermally treated at 80 or 90 °C at different times in a closed container to avoid water evaporation.

#### 3.2.2. Preparation of Ag Nanoparticles in chondroitin Sulfate Solution by UV-Irradiation

First 400 µL of AgNO_3_ (1 M) dissolved in water were added to chondroitin sulfate (10% *w*/*v*) and then the mixture was irradiated (λ = 254 nm) at different times in a home-made dark chamber. The distance between the lamp and the sample was fixed at 23 cm.

#### 3.2.3. Preparation of AgNPs-Chondroitin Sulfate and AgNO_3_-Chondroitin Sulfate Scaffolds

To produce AgNPs-CS and AgNO_3_-CS scaffolds, insulin syringes were filled with the polymer solutions (irradiated or non-irradiated) containing the AgNPs or AgNO_3_. The syringes were unidirectionally frozen by immersion into a liquid nitrogen bath (77 K) at constant rate (2.7 mm/min). The frozen samples were freeze-dried using a Thermo Savant Micromodulyo freeze-drier. The resulting samples were monoliths with the geometry of the insulin syringes. Monoliths were further crosslinked using hexamethylene diisocyanate (HDI) vapors. Briefly, the scaffolds were exposed to HDI vapors in a closed container at 60 °C during one week. After that, the samples were aerated during 48 h. Non-crosslinked monoliths were also produced.

#### 3.2.4. Sample Characterization

Monoliths morphologies were evaluated by scanning electron microscopy (Zeiss DSM-950 instrument (Zeiss, Oberkochen, Germany). The morphologies and size distribution of AgNPs were studied by electron transmission microscopy (200 KeV JEOL 2000 FXII; JEOL, Tokyo, Japan). The selected area electron diffraction (SAED) was used to study the crystallinity. UV/Vis spectrometry analyses were performed in a Variant Cary 4000 spectrophotometer (Agilent, Santa Clara, CA, USA) in the wavelength range of 350–600 nm by step of 1 nm. X-ray photoelectron spectroscopy surface (XPS) analysis was carried out to determine the ionized state of silver. Samples were analyzed using a VG ESCALAB 200R electron spectrometer (Thermo Fisher Scientific Inc, Waltham, MA, USA). The equipment is outfitted with a hemispherical electron analyzer (Pass energy: 50 eV) and an Al Kα 120 W X-ray source. The binding energies were referenced to the binding energy of C1s core-level spectrum at 284.9 eV. Data were acquired and processed using Avantage software provided by the manufacturer. Shirley background was subtracted previously to spectra decomposition with the least-squares fitting routine. To determine atomic fraction, peak areas normalized on the basis of sensitivity factors provided by the manufacturer were used.

#### 3.2.5. Silver Release

The release of the AgNPs from the AgNPs-CS crosslinked scaffolds in PBS was evaluated. A known amount of the monolith (ca. 12 mg) was put in 1 mL of PBS at 37 °C. The solvent was completely removed at different times (1, 2, 3, 4, 6 and 24 h) and fresh PBS was added. The removed PBS solution was dissolved in HNO_3_ 0.1 M and filtered through a 0.45 mm regenerated cellulose filter (Albet, Relliehausen, Germany). The silver content was determined by inductively coupled plasma mass spectrometry using a OPTIMA 2100 DV spectrometer (PERKIN ELMER, Waltham, MA, USA).

#### 3.2.6. Antibacterial Activity

To determine the minimum inhibitory concentration (MIC) of the AgNPs included in CS scaffolds, we used a known amount of non-crosslinked scaffolds with a known silver content. The AgNPs-CS scaffold was dissolved in 1 mL PBS to completely release the AgNPs included in the scaffold and dilutions were carried out in PBS. As control a non- crosslinked CS scaffold was also dissolved in PBS (sample denoted as 0 ppm). Each solution was added to bacteria culture medium (with a final AgNPs concentration between 0 to 6.25 ppm) and the absorbance at 600 nm was measured during 6 h after bacterial inoculation. To evaluate the antibacterial properties of the scaffolds, *E. coli* TG1 was grown at 37 °C during 24 h in aerated flasks containing Lysogeny broth (LB) medium in the presence of AgNPs-CS crosslinked scaffolds, AgNO_3_-CS crosslinked scaffolds and CS cros-slinked scaffolds (control without silver). AgNO_3_ aqueous solutions at 6.25 and 12.5 ppm were also tested. For comparative purposes, the same amount of scaffold (ca. 60 mg) containing around 285 µg of silver per gram of polymer was used in each experiment.

## Abbreviations

The following abbreviations are used in this manuscript:

AgNPs	Silver nanoparticles
CS	Chondroitin sulphate
TEM	Transmission electron microscopy
MWCNTs	Multi-walled carbon nanotubes
XPS	X-ray photoelectron spectroscopy
SAED	selected-area electron diffraction
ISISA	ice-segregation-induced self-assembly
SEMPBS	Scanning electron microscopyPhosphate saline buffer
MIC	Minimum inhibitory concentration
LB	Lysogeny broth medium
HDI	Hexamethylenediisocyanate
